# Case Report: Faricimab in the treatment of subretinal fluid secondary to choroidal osteoma without definite evidence of CNV

**DOI:** 10.3389/fmed.2026.1973913

**Published:** 2026-09-18

**Authors:** Ruixue Ren, Yulu He, Yuhan Shang, Yilan Hu, Chaoqun Liu, Xiaoli Yang

**Affiliations:** 1 Department of Ophthalmology, Affiliated Hospital of North Sichuan Medical College, Nanchong, Sichuan, China; 2 North Sichuan Medical College, Nanchong, Sichuan, China; 3 Department of Ophthalmology, The Second Clinical College of North Sichuan Medical College, Nanchong Central Hospital, Nanchong, Sichuan, China

**Keywords:** anti-VEGF therapy, choroidal osteoma, faricimab, subretinal fluid, vascular endothelial growth factor

## Abstract

**Background:**

Choroidal osteoma is a rare benign osseous tumor that most commonly occurs in young women, typically located in the choroid near the optic disc or in the macular region. Secondary choroidal neovascularization (CNV) or subretinal fluid (SRF) is the main causes of visual impairment in affected patients.

**Case report:**

This article reports a 36-year-old woman who presented with decreased vision in her right eye. Multimodal imaging confirmed a choroidal osteoma in the right eye complicated by SRF in the macular area. After intravitreal injections of ranibizumab, SRF decreased substantially. Approximately 40 weeks later, the patient reported recurrent visual decline, and macular SRF recurs. After switching to intravitreal injection of faricimab (6 mg/0.05 mL), the SRF resolved completely, macular complete SRF resolution with restoration of the foveal contour, and visual acuity remained stable throughout subsequent follow-up.

**Conclusion:**

This case suggests that anti-VEGF therapy may be an option for SRF secondary to choroidal osteoma. In recurrent cases without definite CNV, faricimab may facilitate SRF resolution, though the proposed Ang-2-mediated vascular-stabilizing effect requires further validation.

## 1 Introduction

Choroidal osteoma is a rare benign osseous tumor that occurs predominantly in young women and typically arises in the choroid near the optic disc or macular area (1). In 1978, Gass et al. first systematically described the clinical and pathological features of this disease (2). Choroidal osteoma frequently leads to secondary choroidal neovascularization (CNV), resulting in leakage, hemorrhage, and subretinal fluid (SRF), which represent major causes of visual impairment (3, 4). In addition to photodynamic therapy (PDT) and laser photocoagulation, intravitreal anti-vascular endothelial growth factor (anti-VEGF) therapy is now widely used to manage complications such as CNV (5). However, a substantial proportion of cases present with serous retinal detachment without definite evidence of neovascularization, which may reflect breakdown of the blood-retinal barrier secondary to decompensation and degeneration of the retinal pigment epithelium (RPE) overlying the tumor, as well as tumor decalcification (6–8). Marked heterogeneity exists in the response to anti-VEGF therapy for SRF secondary to choroidal osteoma (6, 9). Previous studies have shown that even when initial treatment is effective, up to 70.6% of treated eyes experience recurrence within 1 year (6), and late recurrence following anti-VEGF therapy remains a major clinical management challenge.

Faricimab is a novel bispecific antibody that simultaneously inhibits vascular endothelial growth factor A (VEGF-A) and angiopoietin-2 (Ang-2). Through this dual mechanism, it suppresses vascular leakage while promoting vascular stability (10). Faricimab has demonstrated favorable therapeutic efficacy in diseases such as neovascular age-related macular degeneration and diabetic macular edema (11, 12); however, reports describing its use in choroidal osteoma remain scarce.

This case of choroidal osteoma showed initial SRF resolution with ranibizumab but recurrence after withdrawal. Switching to faricimab achieved complete SRF resolution and stable BCVA, with no recurrence observed during follow-up.

## 2 Case report

A 36-year-old woman presented to our hospital with a 1-month history of decreased vision in the right. She denied any history of ocular disease, trauma, inflammatory disease, or hereditary ocular conditions. Her best-corrected visual acuity (BCVA) was 20/50 in the right eye and 20/20 in the left eye. Intraocular pressures were 14 mmHg in both eyes (1 mmHg = 0.133 kPa).

On examination, both eyes showed no abnormality in the anterior segment. Ocular movements in all directions were normal. Color fundus photography of the right eye revealed an approximately 5-disc-diameter-sized arc-shaped orange-yellow elevated lesion with well-defined borders above the optic disc. The lesion extended in a pseudopod-like manner along the inferior margin of the optic disc, with macular edema and yellow-white hard exudates observed (Figure 1A). Spectral-domain optical coherence tomography (SD-OCT) of the lesion area showed a locally elevated “spongiform” high-reflectivity pattern in the subretinal space, with scattered punctate high-reflectivity signals between retinal layers. The retinal pigment epithelium (RPE) layer and the ellipsoid zone were atrophic and disorganized, and continuity of the outer retinal layer was interrupted (Figure 1B). Obvious SRF was present in the macula. Only a few punctate high-reflectivity signals were observed between retinal layers, with no definite evidence of CNV such as pigment epithelial detachment, subretinal hyperreflective material, or irregular RPE elevation (Figure 1C). Infrared imaging (IR) showed the lesion outline (Figure 2A). Fluorescein fundus angiography (FFA) showed patchy hyperfluorescence in the early phase (Figure 2B), In the late phase, fluorescence slightly diminished without definitive focal leakage, although occult CNV cannot be entirely ruled out. Indocyanine green angiography (ICGA) revealed that the main body of the tumor showed persistent hypofluorescence (Figure 2C). Ocular ultrasonography A-scan showed high-intensity reflectance peaks at the lesion site. B-scan showed an isolated, low-elevated strong-echo focus near the optic disc, with obvious posterior acoustic shadowing; the post-bulbar tissue was obscured (Figure 2D). Magnetic resonance imaging (MRI) T1-weighted image of the right eye; the green arrow points to a small nodular hypointense lesion at the optic disc (Figure 2E). Orbital computed tomography (CT) showed an arc-shaped hyperdense shadow was seen along the posterior wall of the globe in the right eye, consistent with bony density, measuring approximately 0.7 cm × 0.5 cm, with a CT value of approximately 300 HU, the lesion is indicated by the red arrow (Figure 2F).

**Figure 1:**
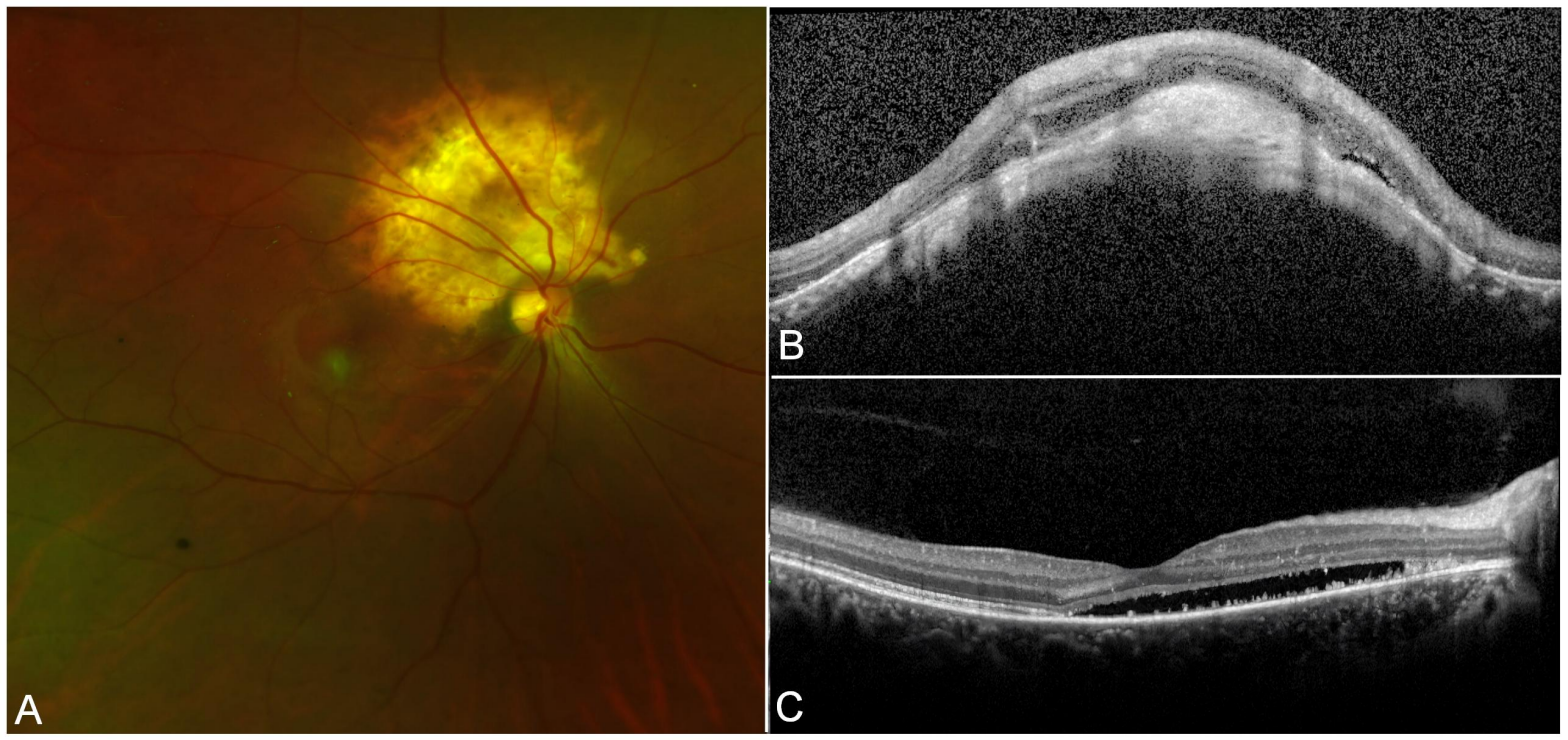
Color fundus photograph and SD-OCT images of the right eye. **(A)** Color fundus photograph showing an orange-yellow lesion above the optic disc with macular exudation. **(B)** Baseline SD-OCT showing a tumor-like mass elevated above the optic disc with destruction of the retinal structure. **(C)** SD-OCT showing subretinal fluid (SRF) accumulation in the macular area, accompanied by punctate hyperreflective signals in the intraretinal layers and disruption of the ellipsoid zone. No definite evidence of CNV such as pigment epithelial detachment, subretinal hyperreflective material, or irregular RPE elevation.

**Figure 2:**
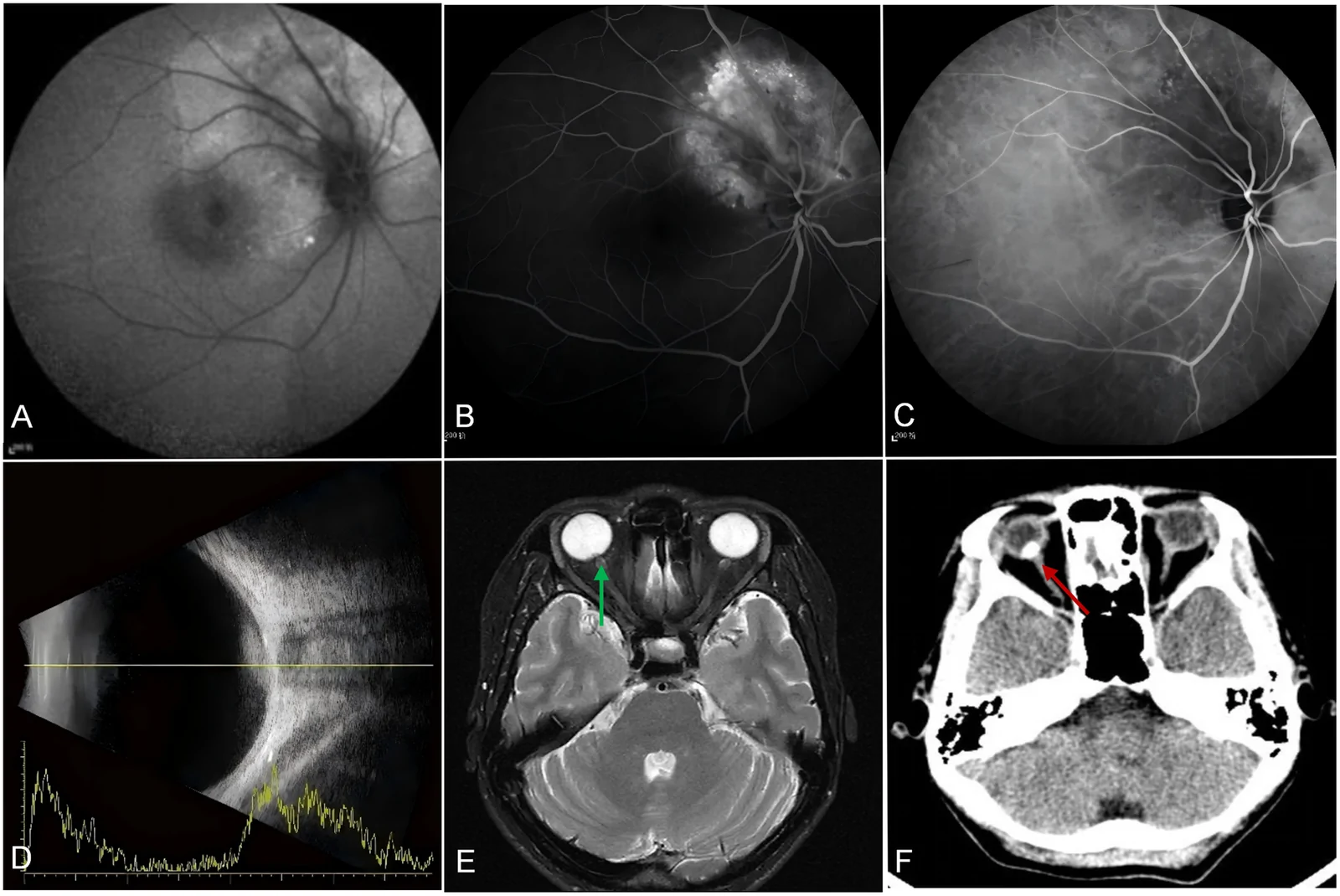
Multimodal imaging of the right eye. **(A)** Infrared imaging (IR) showing the lesion outline. **(B)** Fluorescein fundus angiography (FFA) showing the speckled high fluorescence in the early phase. **(C)**Indocyanine green angiography (ICGA) showing that the lesion had low fluorescence. **(D)** Ocular ultrasonography A-scan showed high-intensity reflectance peaks at the lesion site. B-scan showed an isolated, low-elevated strong-echo focus near the optic disc, with obvious posterior acoustic shadowing; the post-bulbar tissue was obscured. **(E)** MRI T1-weighted image of the right eye; the green arrow points to a small nodular hypointense lesion at the optic disc. **(F)** Bilateral orbital CT images, a red arrow points to a curved high-density bony shadow along the posterior wall of the globe in the right eye.

The patient received intravitreal injections of ranibizumab (0.05 mL, 10 mg/mL) per monthly for three consecutive months. After completing the three injections during the loading period, the best visual acuity (BCVA) of the right eye improved to 20/25. SD-OCT showed that macular SRF had decreased substantially compared with baseline but had not resolved completely; a small amount of residual SRF persisted, presenting as shallow serous retinal detachment (Figure 3). *given the limitations inherent* Approximately 40 weeks after the first ranibizumab injection, the patient reported recurrent visual decline in the right eye, accompanied by worsening of the perceived dark shadow. Right eye BCVA decreased to 20/50, Optical coherence tomography angiography (OCTA) showed no signs of active CNV (Figure 3A). SD-OCT showed macular SRF recurred substantially, approaching the severity observed at initial presentation (Figure 3B). Given the incomplete anatomic response to ranibizumab (failure to achieve complete SRF resolution) and late recurrence following initial functional improvement, treatment was switched to intravitreal faricimab (6 mg/0.05 mL), administered once monthly for three consecutive months. Twelve weeks after the first faricimab injection—that is, after completion of the loading-phase treatment—right eye BCVA improved to 20/20. SD-OCT demonstrated complete resolution of SRF in the macula, with restoration of foveal contour and retinal layer architecture (Figure 4E). At the last visit, 32 weeks after switching to faricimab, right eye BCVA remained at 20/20, and OCT showed that the macula remained dry throughout the follow-up period, OCTA showed no obvious CNV (Figure 4F). No ocular or systemic adverse events occurred during treatment or follow-up, and no abnormalities were observed in the anterior segments of either eye. The clinical course and imaging manifestations during the treatment and follow-up period are shown in Table 1. This case report adhered to the principles of the Declaration of Helsinki; treatment and publication were performed with the patient's written informed consent, and all patient data were fully de-identified to ensure anonymity. This report complies with the CARE (Case Report) guidelines.

**Figure 3:**
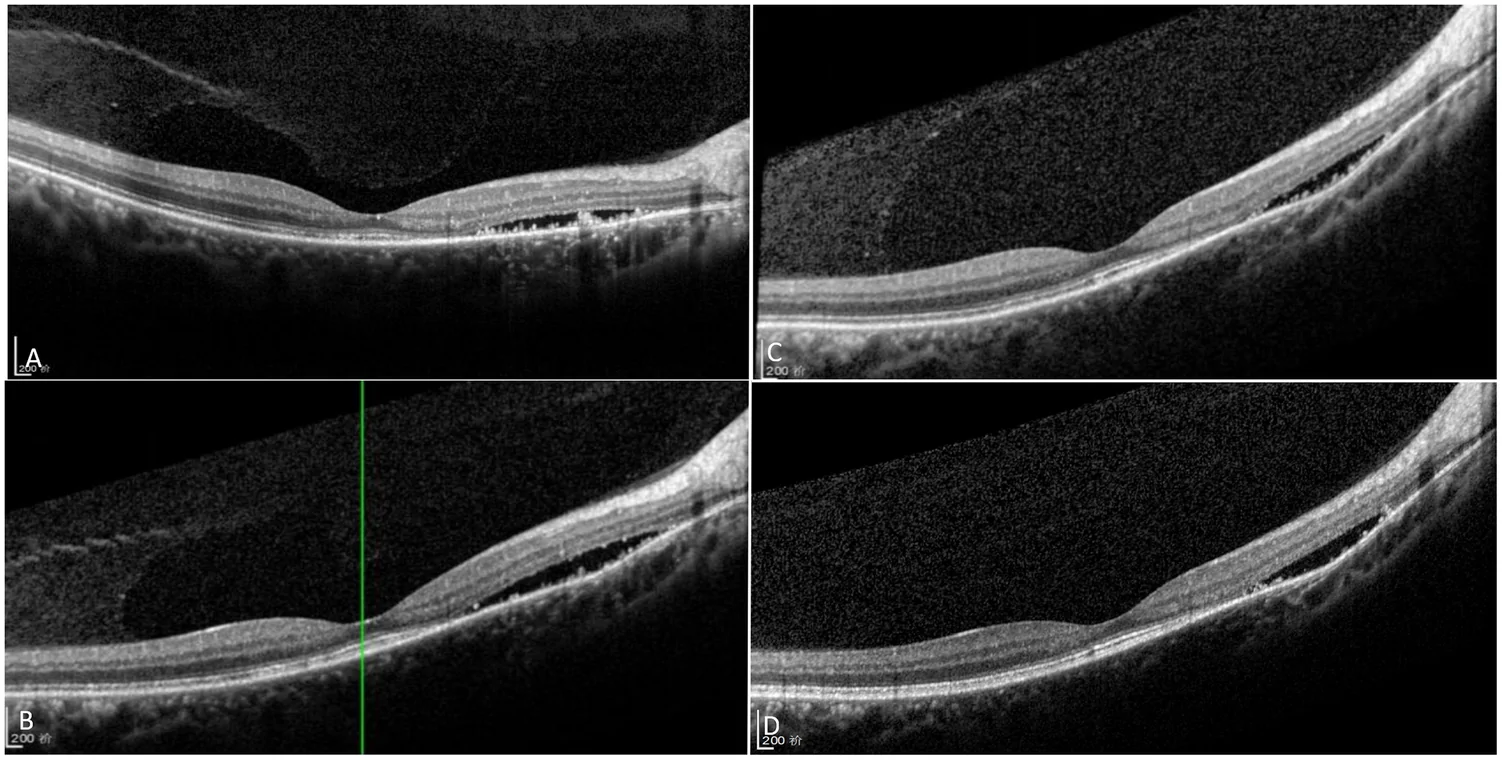
Right eye SD-OCT images. **(A)** SRF at 4 weeks after the first ranibizumab injection (4 weeks post-baseline); **(B)** SRF at 8 weeks post-baseline after the second ranibizumab injection; **(C)**SRF at12 weeks after the third ranibizumab injection. Compared with baseline, macular SRF is substantially reduced, but a small amount of residual SRF persists, presenting as shallow serous retinal detachment. Anti-VEGF therapy was subsequently paused, and the patient entered a period of clinical observation. **(D)** During the observation period (28 weeks post-baseline), a small amount of residual SRF persists.

**Figure 4:**
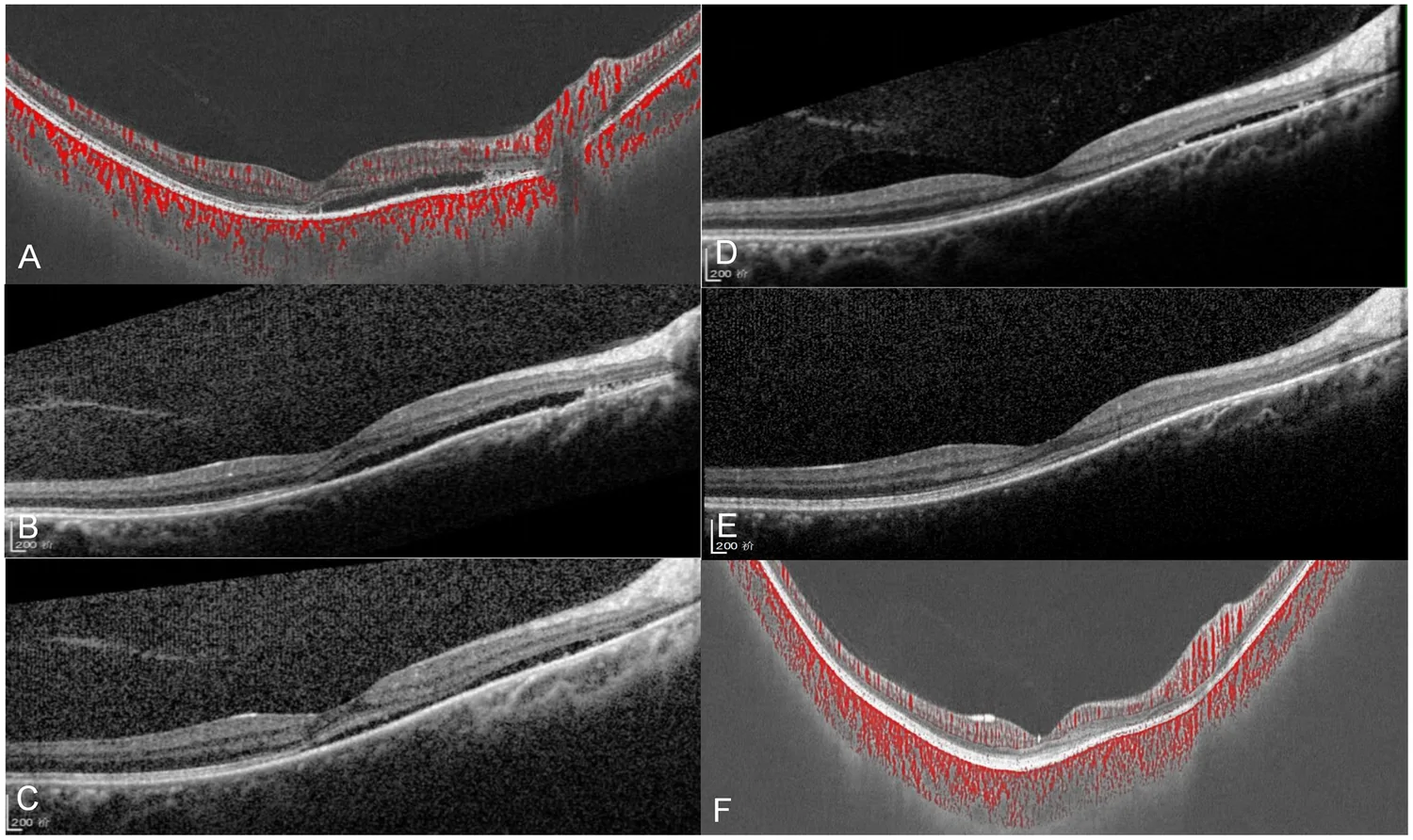
Right eye OCTA and SD-OCT images. **(A)** Forty weeks after the first ranibizumab injection, optical coherence tomography angiography (OCTA) showed No signs of active CNV flow signal were observed. **(B)** At 40 weeks, OCT showed substantial recurrence of macular subretinal fluid (SRF), approaching baseline severity; **(C)** SRF at 4 weeks after the first faricimab injection. **(D)** SRF at 4 weeks after the second faricimab injection. **(E)** at 4 weeks after the third faricimab injection, complete resolution of macular SRF with restoration of foveal contour and retinal layer architecture. **(F)** At the final follow-up visit (72 weeks after baseline/ 32 weeks after the first faricimab injection), OCTA shows no obvious CNV.

**Table 1:** Clinical course and imaging findings during treatment and follow-up.

Follow-up date	Event	Visual acuity (Right eye)	OCT/CRT
Row 1: Baseline (0 wk)	1st Ranibizumab	20/50	SRF accumulation, intraretinal punctate hyperreflective foci, disruption of the ellipsoid zone, CRT: 231 μm
Row 2: 4 wk	2nd Ranibizumab	20/30	Residual SRF，CRT: 176 μm
Row 3: 8 wk	3rd Ranibizumab	20/25	Residual SRF，CRT: 171 μm
Row 4: 12 wk	Clinical observation period	20/25	Residual SRF persisted, shallow serous retinal detachment CRT: 175 μm
Row 5: 28 wk	Clinical observation period	20/25	Residual SRF persisted, shallow serous retinal detachment CRT: 184 μm
Row 6: 40 wk	1st Faricimab	20/50	SRF approaching baseline severity CRT: 223 μm
Row 7: 44 wk (4 wk after 1st Faricimab)	2nd Faricimab	20/30	SRF reduced CRT: 175 μm
Row 8: 48 wk (8 wk after 1st Faricimab)	3rd Faricimab	20/20	Reduction of SRF and inter-layer high reflection signals CRT: 164 μm
Row 9: 52 wk (12 wk after 1st Faricimab/4 wk after 3rd Faricimab)	Clinical observation period	20/20	complete SRF resolution with restoration of the foveal contour CRT: 168 μm
Row 10: 72 wk (32 wk after 1st Faricimab)	Last visit	20/20	No recurrence of SRF was observed. CRT: 164 μm

## 3 Discussion

Choroidal osteoma is composed predominantly of mature bony trabeculae and a vascularized marrow cavity (2). The tumor typically arises in the choroid near the optic disc or macular area and presents as a yellow-white or orange-red, flat, elevated lesion (13). Multimodal imaging is essential for confirming the diagnosis of choroidal osteoma. In this case, color fundus photography revealed an arc-shaped, orange-yellow elevation above the optic disc; ultrasonography demonstrated a hyperechoic focus with posterior acoustic shadowing; and CT identified a lesion with bony density. Together, these findings provided robust evidence supporting the diagnosis of choroidal osteoma.

Visual impairment in choroidal osteoma results primarily from direct destruction of the RPE and photoreceptor layer by the tumor, as well as from secondary SRF or CNV (8). In this case, OCT and angiographic findings showed no definite evidence of active CNV, suggesting that the SRF was not attributable typical CNV-related leakage. However, the presence of occult choroidal neovascularization cannot be ruled out.

Choroidal osteoma-associated SRF is often characterized by a chronic course, frequent recurrence, and marked interindividual variability. Large case series have demonstrated that although SRF may resolve after treatment in some eyes, recurrence is not uncommon, often necessitating repeated injections and long-term management (3). Long-term outcomes are further influenced by factors such as tumor thickness, outer retinal damage, and fibrosis (6, 14, 15). Longitudinal follow-up data suggest that after an initial course of three injections, the condition may remain stable for years; however, new CNV and exudation may subsequently develop, requiring further treatment (16). Large sample studies indicate that the rate of complete or partial response to anti-VEGF therapy for SRF without definite evidence of CNV is lower than that observed in eyes with CNV, suggesting that the pathological basis of this type of exudation is more complex and the treatment response more limited (6).

Anti-VEGF therapy represents an important intervention for choroidal osteoma-associated SRF. Many symptomatic cases of fluid accumulation are related to active neovascularization, with the VEGF pathway playing a central role in vascular leakage, increased permeability, and maintenance of abnormal vessels (3, 17). In patients without definite evidence of CNV, prior small case series and reports have shown that anti-VEGF agents such as ranibizumab and bevacizumab can effectively promote resolution of isolated SRF, improve retinal anatomy, and provide a degree of visual stabilization or improvement (3, 4, 6). In case series of serous retinal detachment, even when CNV was clearly present in only one eye, other eyes without definite CNV also demonstrated visual improvement and reduced central foveal thickness following intravitreal bevacizumab, suggesting that anti-VEGF therapy may also be effective for atypical or occult leakage (7). In our case, SD-OCT demonstrated SRF, while angiography and OCTA showed no definite evidence of CNV. Following the initial intravitreal injection of ranibizumab, SRF decreased substantially and visual acuity improved to 20/20, supporting the effectiveness of anti-VEGF agents for isolated SRF associated with choroidal osteoma. Treatment was paused after three ranibizumab injections despite residual SRF, according to the patient's preference and clinical observational strategy. It remains unclear whether the residual SRF corresponds to chronically stable fluid, a delayed response to ranibizumab, or genuine treatment failure.

Faricimab is the first bispecific antibody approved for intraocular use, simultaneously inhibiting VEGF-A and ANG-2, thereby suppressing vascular leakage while promoting vascular stability through a dual mechanism (10). In neovascular age-related macular degeneration, faricimab has been associated with more effective and durable control of intraretinal fluid (IRF) and SRF compared with agents targeting VEGF-A alone, along with a shorter time to first resolution of retinal fluid (18). Recent systematic reviews and pooled analysis have shown that the use of faricimab for wet agerelated macular degeneration has resulted in improved macular anatomy (19). A recent report by Fadwa Al Adel and colleagues described a patient with choroidal osteoma who had persistent intraretinal and subretinal fluid despite treatment with ranibizumab and aflibercept; after switching to faricimab, complete fluid resolution was achieved, allowing extension of the treatment interval to 16 weeks (20). In our case, we elected to switch to faricimab at the time of SRF recurrence. Following three loading doses, complete resolution of SRF and restoration of normal foveal architecture were achieved, and no recurrence was detected throughout the 32-week follow-up period.

However, faricimab treatment was temporally associated with SRF resolution. Due to the nonrandomized crossover and different disease phases, superiority over ranibizumab cannot be concluded. Further studies are warranted to elucidate the potential role of dual-targeted anti-VEGF agents in this rare entity.

## 4 Conclusion

This single case observation suggests that intravitreal faricimab may be temporally associated with the resolution of recurrent SRF secondary to choroidal osteoma in the absence of detectable active CNV. However, given the limitations inherent to a single case observation, these findings require further confirmation through accumulation of additional cases and larger sample studies employing standardized outcome measures and longer follow-up periods, in order to comprehensively evaluate the therapeutic efficacy and safety of faricimab in choroidal osteoma-related exudation.

## Data Availability

The original contributions presented in the study are included in the article/Supplementary Material, further inquiries can be directed to the corresponding author/s.
